# Molecular Mechanisms of *Trichosanthes kirilowii* in Response to Root-Knot Nematode Infection

**DOI:** 10.3390/ijms27031594

**Published:** 2026-02-06

**Authors:** Lei Zheng, Huadong Wang, Zhiqiang Zhang, Jiuling Gu

**Affiliations:** 1 School of Pharmacy, Sichuan College of Traditional Chinese Medicine, Mianyang 621000, China; 2 Northwest Sichuan Laboratory of Traditional Chinese Medicine Resources Research and Development Utilization, Mianyang 621000, China; 3 Mianyang Key Laboratory of Development and Utilization of Chinese Medicine Resources, Mianyang 621000, China

**Keywords:** *Trichosanthes kirilowii*, root-knot nematode, infection, response, transcriptome

## Abstract

Root-knot nematode (RKN) infection poses a serious threat to the yield and quality of the perennial medicinal plant *Trichosanthes kirilowii* (*T. kirilowii*) in cultivation. However, its response mechanisms remain unclear. This study analyzed root growth and transcriptomic data at various root-knot nematode infection time points (0, 3, 6, 12, and 24 days post-infection, dpi) for *T. kirilowii* to reveal its specific response mechanisms. The results showed that RKN infection significantly affected the root growth and gene expression of *T. kirilowii*. At 24 dpi, individual plants formed an average of 69 galls, indicating the plant shows susceptibility. A total of 14,243 differentially expressed genes (DEGs) were identified, including 382 transcription factors. Weighted gene co-expression network analysis (WGCNA) of DEGs identified four key modules closely associated with RKN infection. GO and KEGG enrichment analyses indicated multiple metabolic pathways involved in the response process, including defense responses, hormone signaling, phenylpropanoid biosynthesis, and MAPK signaling pathways. Hub gene analysis of key modules identified 33 hub genes, including three critical transcription factors. This study demonstrates that *T. kirilowii* responds to RKN infection through coordinated regulation of multiple metabolic pathways and transcriptional regulatory networks. These findings enhance understanding of the molecular mechanisms underlying *T. kirilowii*–RKN interactions and provide critical insights for further research on resistance mechanisms and the identification of resistance genes in *T. kirilowii*.

## 1. Introduction

RKNs pose a severe threat to numerous important crops and cause significant yield losses or even complete crop failure [1,2,3]. It is estimated that RKNs inflict approximately USD 150 billion in economic losses worldwide annually, presenting a long-term and severe challenge to food security and agricultural economic stability [4,5]. Common RKN species include *Meloidogyne incognita*, *Meloidogyne hapla*, *Meloidogyne javanica*, and peanut RKN [6,7]. The second-stage juveniles (J2) infect roots, forming galls that disrupt root structure and function. This impairs water and nutrient uptake, leading to stunted growth and reduced yield and quality [6,8,9]. Furthermore, nematode-induced root wounds facilitate infection by soil-borne fungi and bacteria, triggering compound diseases and increasing crop loss [6,10,11,12]. Currently, chemical control remains the primary method for managing RKN diseases. However, prolonged pesticide reliance causes environmental pollution, residue issues, and increasing nematode resistance [13,14]. Therefore, developing effective and eco-friendly control strategies, especially by exploring plants’ inherent disease-resistance genetic resources, is an urgent and critical research direction for RKN management.

The plant–RKN interaction involves a complex molecular network. High-throughput sequencing, especially transcriptomics, has recently enabled deeper investigation into these mechanisms [15,16]. Studies indicate that RKNs secrete numerous effector proteins into plant cells through their stylets to suppress basal immune responses and establish feeding sites [17,18]. In response, plants activate PAMP-triggered immunity (PTI) through pattern recognition receptors (PRRs) and establish a multi-layered defense system via effector-triggered immunity (ETI) mediated by resistance proteins, as well as hormonal signaling pathways involving salicylic acid (SA), jasmonic acid (JA), and ethylene (ET) [19,20]. Numerous plant genes differentially expressed following an RKN infection have been identified, including key genes associated with reactive oxygen species (ROS) bursts, calcium signaling, cell wall reinforcement, and phenylpropanoid metabolism. These discoveries have significantly enriched our understanding of plant nematode resistance mechanisms and provided molecular targets for breeding resistance to RKNs [21,22,23,24]. Despite this progress, research has largely focused on model and vegetable crops like tomatoes and Arabidopsis. Studies on medicinal plants, particularly *Trichosanthes kirilowii*, remain limited and urgently need strengthening.

*Trichosanthes kirilowii* Maxim. is a perennial medicinal plant belonging to the Cucurbitaceae family. Its fruit, seeds, peel, and tuberous roots are all used medicinally, known, respectively, as Gualou, Gualou Seeds, Gualou Peel, and Tianhuafen [25]. *T. kirilowii* is widely distributed across Chinese provinces, including Anhui, Sichuan, Shandong, Henan, Hebei, and Jiangsu. It is commonly used to treat angina pectoris, chest tightness, constipation, and pulmonary heart disease and finds extensive applications in the pharmaceutical, food, and cosmetics industries, possessing high economic and medicinal value [25,26,27]. Due to robust market demand, numerous cultivation bases have been established in China. However, *T. kirilowii* cultivation frequently suffers from RKN infections, which can severely reduce yields or even cause complete crop failure, posing a significant threat to its industrial development [28]. Current management of *T. kirilowii* RKN disease primarily relies on chemical pesticides, which are costly and often ineffective. Therefore, there is an urgent need to explore resistant variety breeding programs to effectively address this critical issue. Despite expanding cultivation areas, research on RKN diseases in *T. kirilowii* remains relatively limited. This study focuses on the response of *T. kirilowii* to RKN infection, with the goal of characterizing the temporal association between phenotypic and transcriptomic changes, as well as the core metabolic and signaling pathways involved, and identifying key co-expression modules and hub genes to elucidate the molecular mechanisms underlying the *T. kirilowii*–RKN interaction.

## 2. Results

### 2.1. Phenotypic Changes of T. kirilowii Root in Response to RKN Infection

**Figure 1:**
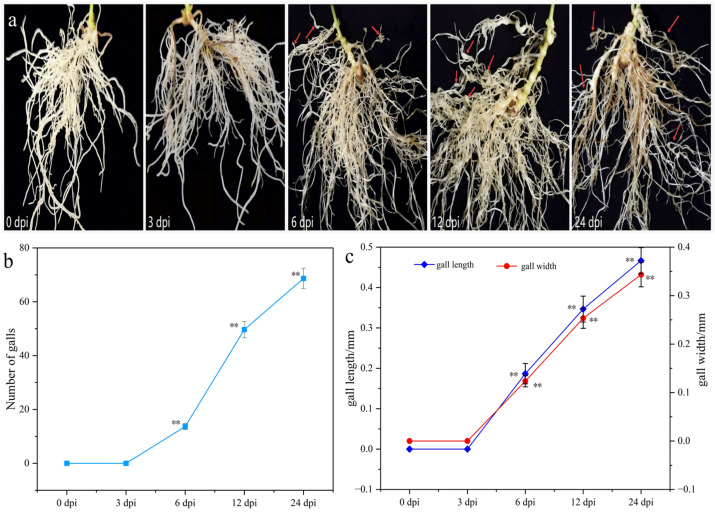
Phenotypic changes of *T. kirilowii* root in response to RKN infection. (**a**) Root growth trait. The red arrows point to the galls. (**b**) Number of galls. (**c**) Gall length and width. Data are presented as the mean ± standard deviation, and triplicate biological replicates were performed. **, *p* < 0.01.

Phenotypic changes of *T. kirilowii* root were observed at 0, 3, 6, 12, and 24 days post-infection (dpi) with RKNs. The results showed (Figure 1) that no obvious symptoms appeared in roots at 3 dpi. At 6 dpi, localized root swelling began to appear in a small number of roots, with an average of 14 galls per plant and with the gall length and width measuring only 0.18 mm and 0.12 mm, respectively. At 12 dpi, distinct galls had formed with significantly increased numbers, reaching an average of 50 galls per plant, and the gall length and width increased to 0.35 mm and 0.25 mm. At 24 dpi, the number of galls further increased, individual gall size enlarged, and some roots began yellowing and senescing. At this stage, the number of galls per plant reached 69, with the gall length and width increasing to 0.47 mm and 0.34 mm, respectively. The significant increases in gall number, gall length, and gall width were temporally consistent with the surge in the number of differentially expressed genes (DEGs) observed at later infection stages (12 and 24 dpi), suggesting a close association between phenotypic deterioration and large-scale transcriptional reprogramming.

### 2.2. Transcriptome Sequence Analysis

To elucidate the response mechanism of *T. kirilowii* to RKN infection, root samples were sequenced at different infection time points. The sequencing results (Table 1) revealed a total of 650,169,152 bp of raw sequences were obtained. After filtering and removing low-quality reads, 646,693,076 clean sequences remained. GC content ranged from 43.93% to 44.59%, with Q30 coverage was between 95.53% and 95.91%. The alignment rate of clean sequences across 15 samples ranged from 86.39% to 87.61%. These findings indicate reliable sequencing data quality.

### 2.3. Analysis of DEGs in T. kirilowii Roots Infected by RKNs at Different Time Points

**Figure 2:**
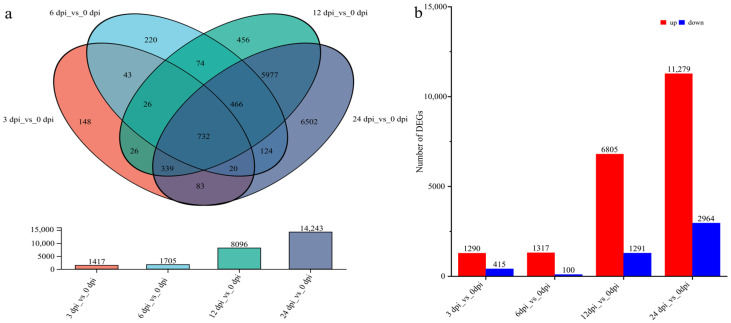
DEGs in *T. kirilowii* roots infected by RKNs at different time points. (**a**) Venn diagram; (**b**) diagram of up-regulated and down-regulated DEG numbers.

Transcriptome analysis of *T. kirilowii* root samples at different RKN infection time points identified DEGs. The results showed (Figure 2a) that the numbers of DEGs at 3 dpi, 6 dpi, 12 dpi, and 24 dpi were 1417, 1705, 8096, and 14,243, respectively. Among these, 148 DEGs were specifically expressed at 3 dpi, 220 at 6 dpi, 456 at 12 dpi, and 6502 at 24 dpi. A total of 732 DEGs were expressed at all five time points. The up-regulation and down-regulation of DEGs statistics showed that compared to 0 dpi, there were 1290, 1317, 6805, and 11,279 up-regulated genes at 3 dpi, 6 dpi, 12 dpi, and 24 dpi, respectively, while the number of down-regulated genes was 415, 100, 1291, and 2964, correspondingly (Figure 2b). The dramatic increase in DEGs at later infection stages (12 and 24 dpi) likely reflects intense and complex transcriptional reprogramming in the host in response to the establishment and maintenance of nematode feeding sites and the associated widespread stress. The overwhelming number of up-regulated genes, particularly at 24 dpi, may be related to the broad activation of host defense-related pathways.

### 2.4. GO Functional Annotation Analysis of DEGs

**Figure 3:**
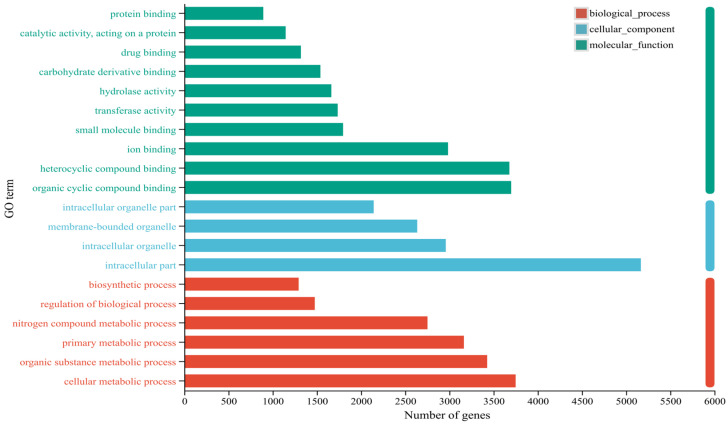
GO functional annotation of DEGs in *T. kirilowii* roots responding to RKN infection.

GO functional annotation analysis of the identified DEGs revealed (Figure 3) that in biological processes (BPs), DEGs were primarily involved in cellular metabolism (3749), organic compound metabolism (3427), primary metabolism (3164), and nitrogen compound metabolism (2752). In cellular components (CCs), DEGs were predominantly involved in intracellular compartments (5166). In molecular functions (MFs), DEGs were primarily associated with organic ring compound binding (3699), heterocyclic compound binding (3679), and ion binding (2984). GO analysis revealed that DEGs were significantly enriched in categories related to stress response, defense response, hormone signal transduction, cytoskeleton organization, and secondary metabolic processes, which are highly consistent with the known plant–nematode interaction mechanisms.

### 2.5. KEGG Pathway Enrichment Analysis of DEGs

**Figure 4:**
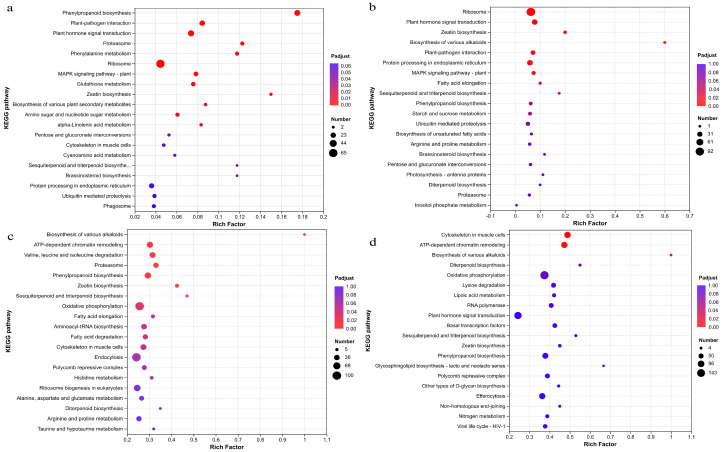
KEGG enrichment analysis of DEGs in *T. kirilowii* roots responding to RKN infection. (**a**) A total of 3 dpi_vs_0 dpi, (**b**) 6 dpi_vs_0 dpi, (**c**) 12 dpi_vs_0 dpi, and (**d**) 24 dpi_vs_0 dpi.

KEGG pathway enrichment analysis of DEGs in *T. kirilowii* roots responding to RKN infection revealed (Figure 4) that DEGs at 3 dpi compared to 0 dpi were significantly enriched in 10 pathways, including the phenylpropanoid biosynthesis, Plant–pathogen interaction, plant hormone signal transduction, Proteasome, Phenylalanine metabolism, Ribosome, MAPK signaling pathway-plant, Glutathione metabolism, Zeatin biosynthesis, and Biosynthesis of various plant secondary metabolites pathways (Figure 4a). The DEGs at 6 dpi compared to those at 0 dpi were significantly enriched in six pathways, including the Ribosome pathway, plant hormone signal transduction pathway, Zeatin biosynthesis pathway, Biosynthesis of various alkaloids pathway, Plant–pathogen interaction pathway, and Protein processing pathways in endoplasmic reticulum (Figure 4b). The DEGs at 12 dpi compared to those at 0 dpi were significantly enriched in six pathways, including the Biosynthesis of various alkaloids; ATP-dependent chromatin remodeling; Valine, leucine, and isoleucine degradation; Proteasome; phenylpropanoid biosynthesis; and Zeatin biosynthesis pathways (Figure 4c). At 24 dpi, the number of enriched KEGG pathways significantly decreased compared to that at 0 dpi; DEGs were significantly enriched in only two pathways, including Cytoskeletons in muscle cells and ATP-dependent chromatin remodeling, which may indicate that the host response had entered a relatively stable phase and transcriptional exhaustion (Figure 4d). KEGG analysis indicated that DEGs were significantly enriched in phenylpropanoid biosynthesis, plant hormone signal transduction, the MAPK signaling pathway, and the Biosynthesis of various alkaloids, which are closely related to feeding site formation or defense signaling.

### 2.6. Transcription Factor Analysis of DEGs

**Figure 5:**
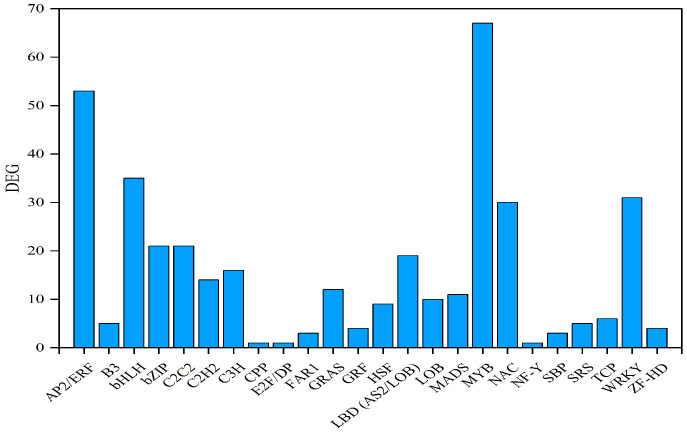
Transcription factor analysis of DEGs in *T. kirilowii* roots responding to RKN infection.

Transcription factor analysis of the identified DEGs revealed (Figure 5) that 382 transcription factors belonging to 24 transcription factor families were observed among the DEGs. The top five transcription factor families were the MYB transcription factor family (67 DEGs), the AP2/ERF transcription factor family (53 DEGs), the bHLH transcription factor family (35 DEGs), the WRKY transcription factor family (31 DEGs), and the NAC transcription factor family (30 DEGs).

### 2.7. Weighted Gene Co-Expression Network Analysis of DEGs

**Figure 6:**
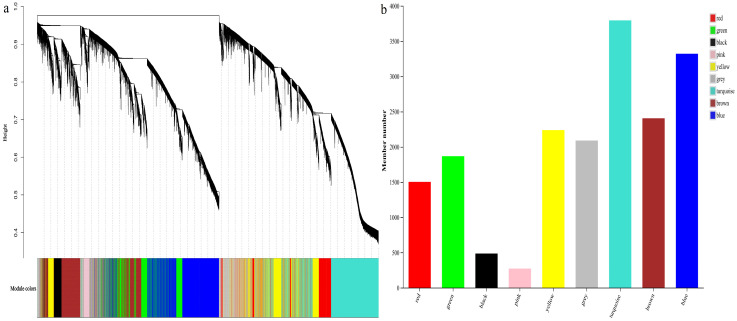
Gene clusters and modules. (**a**) Gene clusters and module color classification; (**b**) number of genes in different colored modules.

DEGs were screened and filtered, excluding low-expression genes (TPM < 1, coefficient of variation < 0.1). A total of 6258 DEGs were obtained for weighted gene co-expression network analysis. Hierarchical clustering of *T. kirilowii* root samples at different infection times revealed two distinct clusters, with no abnormal samples detected. The gene clustering diagram (Figure 6a) revealed seven co-expression modules within the network. The distribution of gene numbers across these modules (Figure 6a) indicated that the turquoise module contained the highest number of DEGs (2548), while the red module had the fewest DEGs (44). The grey module comprised 64 unassigned genes, accounting for 1.02% of the total DEGs.

**Figure 7:**
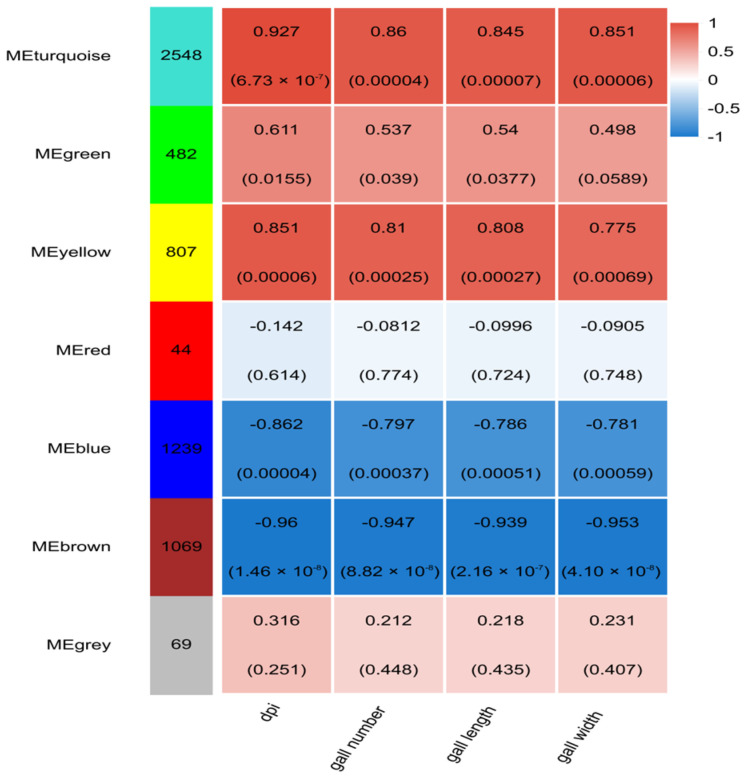
Correlation analysis between modules and root traits.

By calculating correlations between the modules and RKN infection time, gall number, gall length, and gall width, four key modules (MEturquoise, MEyellow, MEblue, and MEbrown) were selected for subsequent analysis based on correlation coefficients and *p*-values (Figure 7). Gall number exhibited significant positive correlation with METurquoise and Meyellow, with correlation coefficients of 0.860 and 0.810, and negative correlation with Meblue and Mebrown, with correlation coefficients of −0.797 and −0.947. Gall length was significantly positively correlated with METurquoise and Meyellow, with correlation coefficients of 0.845 and 0.808, while negatively correlated with Meblue and Mebrown, with correlation coefficients of −0.786 and −0.939. Gall width demonstrated significant positive correlation with METurquoise and Meyellow, with correlation coefficients of 0.851 and 0.775, and negative correlation with Meblue and Mebrown, with correlation coefficients of −0.781 and −0.953. Genes within the MEturquoise and MEyellow modules were significantly up-regulated under RKN infection, while genes in the MEblue and MEbrown modules were significantly down-regulated.

### 2.8. GO Enrichment Analysis of Key Module Genes

**Figure 8:**
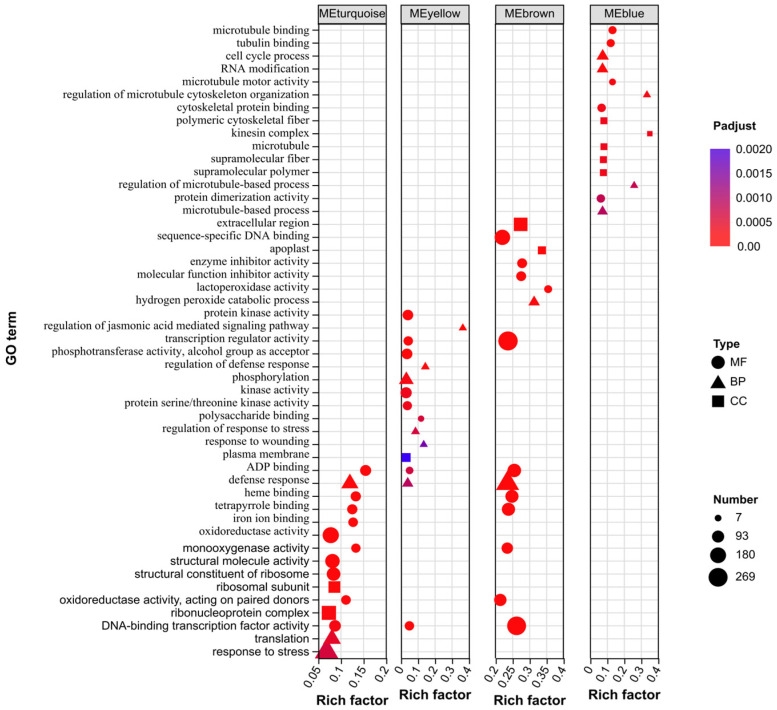
GO enrichment analysis of key module genes.

GO enrichment analysis was performed on the four key module genes, revealing significant functional differences among the modules (Figure 8). In the Biological Process (BP), DEGs in the MEturquoise module were significantly enriched in response to stress, translation, and defense response; DEGs in the MEyellow module showed significant enrichment in phosphorylation, the regulation of defense response, and the regulation of the jasmonic acid-mediated signaling pathway; DEGs in the MEbrown module were significantly enriched in defense response and the hydrogen peroxide catabolic process; DEGs in the MEblue module exhibited significant enrichment in the cell cycle process, RNA modification, and the regulation of microtubule cytoskeleton organization. In the Cellular Component (CC), the MEturquoise module DEGs were significantly enriched in the ribonucleoprotein complex and ribosomal subunit; the MEyellow module DEGs were significantly enriched in the plasma membrane; the MEbrown module DEGs showed significant enrichment in sequence-specific DNA binding; the MEblue module DEGs were significantly enriched in polymeric cytoskeletal fiber, the kinesin complex, microtubules, supramolecular fiber, and supramolecular polymer. In the Molecular Function (MF), the MEturquoise module DEGs were significantly enriched in the oxidoreductase activity, structural molecule activity and structural constituent of ribosome; the MEyellow module DEGs were significantly enriched in kinase activity and phosphotransferase activity, related to alcohol; the MEbrown module DEGs showed significant enrichment in DNA-binding transcription factor activity, transcription regulator activity, and sequence-specific DNA binding; the MEblue module DEGs were significantly enriched in microtubule binding, tubulin binding, and cytoskeletal protein binding.

### 2.9. KEGG Enrichment Analysis of Key Module Genes

**Figure 9:**
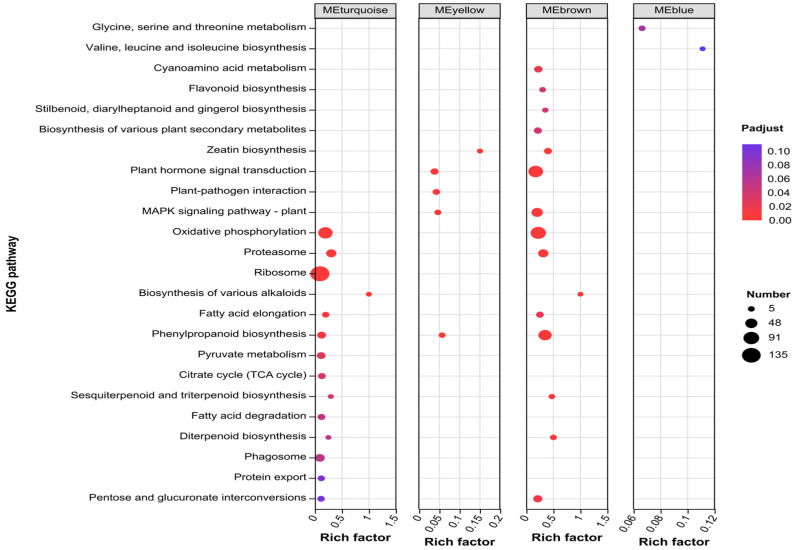
KEEG enrichment analysis of key module genes.

KEGG enrichment analysis of the four key modules revealed (Figure 9) that DEGs in the MEturquoise module were significantly enriched in 12 pathways including the Oxidative, Proteasome, Ribosome, Biosynthesis of various alkaloids, Fatty acid elongation, phenylpropanoid biosynthesis, Pyruvate metabolism, Citrate cycle (TCA cycle), Sesquiterpenoid and triterpenoid biosynthesis, Fatty acid degradation, Diterpenoid biosynthesis, and Phagosome pathways; the MEyellow module DEGs were significantly enriched in 5 pathways including the Zeatin biosynthesis, plant hormone signal transduction, Plant-pathogen interaction, MAPK signaling pathway-plant, and phenylpropanoid biosynthesis pathways; the MEbrown module DEGs were significantly enriched in 15 pathways including the Cyanoamino acid metabolism, Flavonoid biosynthesis, Stilbenoid, diarylheptanoid and gingerol biosynthesis, Biosynthesis of various plant secondary metabolites, Zeatin biosynthesis, plant hormone signal transduction, MAPK signaling pathway-plant, Oxidative phosphorylation, Proteasome, Biosynthesis of various alkaloids, Fatty acid elongation, phenylpropanoid biosynthesis, Sesquiterpenoid and triterpenoid biosynthesis, Diterpenoid biosynthesis, and Pentose and glucuronate interconversion pathways. Notably, the phenylpropanoid biosynthesis pathway was significantly enriched in three key modules (MEturquoise, MEyellow, and MEbrown). Both the plant hormone signal transduction and the MAPK signaling pathway–plant pathways showed significant enrichment in the MEyellow and MEbrown modules. The Oxidative phosphorylation, Proteasome, Biosynthesis of various alkaloids, Fatty acid elongation, Sesquiterpenoid and triterpenoid biosynthesis, and Diterpenoid biosynthesis pathways were all significantly enriched in the MEturquoise and MEbrown modules.

### 2.10. Identification of Hub Genes in T. kirilowii Roots Responding to RKN Infection

The hub genes associated with the response to an RKN infection in *T. kirilowii* roots were identified through calculating the connectivity values of key module genes combined with CytoHubba analysis. A total of 33 hub genes were selected and annotated (Table 2). Specifically, 10 hub genes were identified from the yellow module, 7 from the blue module, 10 from the brown module, and 6 from the turquoise module. Notably, several core genes belong to transcription factor families, including TRINITY_DN4568_c0_g3 (AP2/ERF family), TRINITY_DN9312_c0_g1 (NAC family), and TRINITY_DN2196_c1_g1 (MYB family).

**Figure 10:**
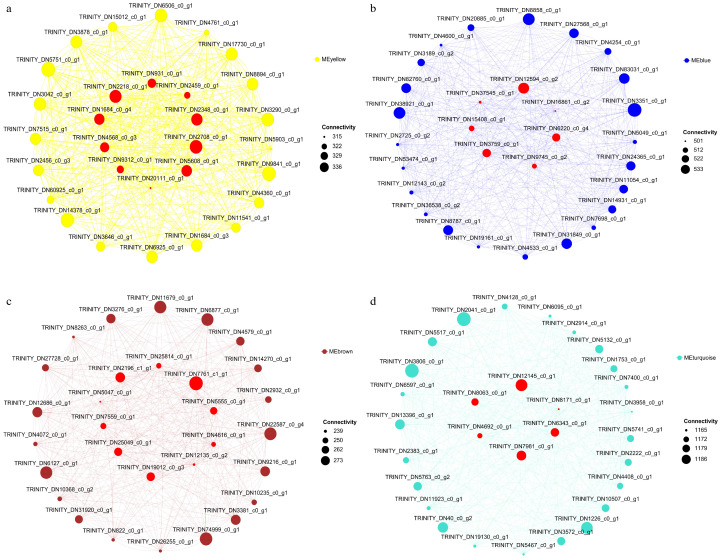
Visual network diagram for hub genes in four modules: (**a**) MEyellow module, (**b**) MEblue module, (**c**) MEbrown module, and (**d**) MEturquoise module. Dots represent genes, red dots represent hub genes, and lines represent connectivity. Dots represent genes, red dots represent hub genes, and lines represent connectivity.

Based on these hub genes and DEGs, the transcriptional regulatory networks of *T. kirilowii* in response to an RKN infection were constructed (Figure 10).

### 2.11. qRT-PCR Validation of DEGs

**Figure 11:**
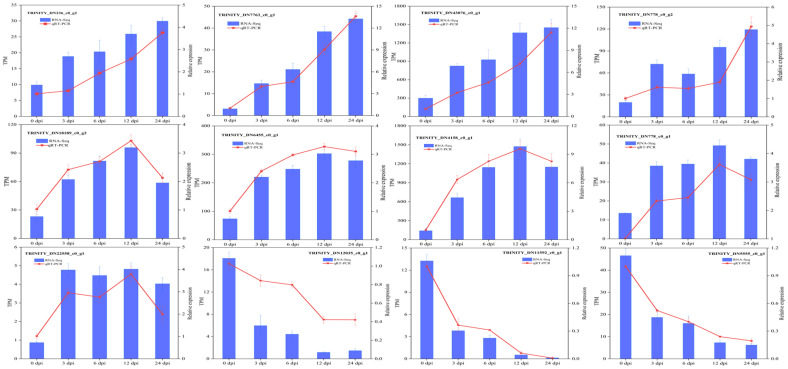
Comparison of RNA-Seq and qRT-PCR expression levels for 12 DEGs. Data are presented as the mean ± standard error, and triplicate biological replicates were performed.

To validate the reliability of the RNA-seq data, 12 significantly DEGs were randomly selected from the DEGs in *T. kirilowii* responding to RKN infection. The expression levels of these genes following RKN infection were analyzed using qRT-PCR technology. The qRT-PCR results (Figure 11) showed that the expression levels and trends of all 12 DEGs were consistent with the RNA-Seq data, indicating a high reliability of the RNA-Seq results.

## 3. Discussion

### 3.1. Time-Dependent Response of T. kirilowii to RKN Infection

The root of *T. kirilowii* exhibits distinct time-dependent characteristics in response to an infection by RKN, manifested as a progressive escalation pattern of symptoms and transcriptional responses. During early infection (3–6 dpi), the root symptoms were mild, with only localized swelling observed and a relatively low number of DGEs (1417–1705). As the infection progressed to 12 dpi, the gall rapidly formed, with the number of DGEs sharply increasing to 8096. By 24 dpi, the gall further expanded, and the number of DGEs surged to 14,243. In summary, the response of T. kirilowii to RKN infection exhibits distinct phasic characteristics. The early stage (3–6 dpi) is characterized by limited symptoms and gene expression changes, reflecting the host’s initial perception and response to the infection; the intermediate stage (12 dpi) is accompanied by rapid root-knot formation and extensive transcriptional reprogramming, indicating the host–nematode interaction has entered an intense phase as the host attempts to initiate multi-level response programs. In the late stage (24 dpi), root galls continued enlarging alongside root senescence, with differentially expressed genes peaking in number. However, the number of significantly enriched KEGG pathways sharply declined, potentially indicating the host response program entered a relatively stable or even exhausted state. This rapid escalation in gene expression dynamics closely paralleled the progression of root symptom development, indicating that *T. kirilowii* employs temporally coordinated multi-layered regulatory mechanisms against the progressive invasion of RKNs. Similar transcriptional response patterns have been observed in various plants, including tomatoes, eggplants, and cucumbers, characterized by a slow onset followed by a dramatic increase in the middle and late stages [29,30,31], suggesting this may represent a conserved response strategy across species. The continuous development and enlargement of galls over time indicate that the *T. kirilowii* in this study exhibits a susceptible phenotype to the RKNs.

### 3.2. Molecular Functional Characteristics of Key Modules

The genes in the MEturquoise module primarily participated in stress response, translation, and defense reactions, indicating extensive metabolic adjustments in the *T. kirilowii* roots during RKN infection, which is consistent with previous findings in rice and tomato crops [29,32]. The genes in the MEyellow module primarily participated in defense responses and jasmonic acid (JA) signaling pathway regulation, carrying multiple transcription factors and signaling proteins with kinase activity. This indicated that signal transduction plays a crucial role in *T. kirilowii*’s response to an RKN infection [33,34,35]. The MEblue module genes were down-regulated during RKN infection, which is potentially linked to the nematode-induced formation of giant cells in vascular sheath cells. Giant cell formation requires altering the normal cell cycle program of host cells, reprogramming them from mitotic mode to nuclear mitosis and cell enlargement mode [36,37]. The MEbrown module genes were enriched in defense responses and hydrogen peroxide (H_2_O_2_) catabolism, showing down-regulation during RKN infection. This down-regulation may contribute to maintaining the short-term survival of infected cells by suppressing apoptosis, thereby enabling the RKNs to fully utilize host resources [38,39,40]. These findings indicated that host molecular responses associated with susceptibility and pathogen counter-defense suppression coexist. The response of *T. kirilowii* to RKN infection involves a complex, multi-layered regulatory process.

### 3.3. Multipathway Coordination Mechanism of T. kirilowii’s Response to RKN Infection

The phenylpropanoid pathway was significantly enriched in three key co-expression modules (MEturquoise, MEyellow, and MEbrown), indicating its central role in defense response. By enhancing phenylpropanoid compound synthesis, the plant roots promote cell wall reinforcement and pathogen barrier formation, thereby restricting the tissue damage caused by RKNs. This mechanism aligns with defense strategies observed in tomatoes and *Pogostemon cablin* [21,41]. Plant hormone signaling pathways and the MAPK signaling cascade were both significantly enriched in the MEyellow and MEbrown modules, indicating coordinated participation of multiple hormone signaling pathways. These findings further confirm that a root-knot nematode infection triggers plant defense responses analogous to those induced by pathogen infections, consistent with the nematode’s role as a biotic stress factor [42,43,44]. The synergistic activation of secondary metabolic pathways, including flavonoid biosynthesis, diterpene biosynthesis, and triterpene biosynthesis, indicates that *T. kirilowii* enhances direct or indirect resistance against RKNs through the accumulation of diverse secondary metabolites. These metabolites not only serve as direct targets for defense molecules but also participate in signaling molecule synthesis. This finding aligns with the results reported by Desmedt et al. [45] in rice and Kirwa et al. [46] in tomato. Despite the activation of extensive defense-related transcriptional programs in *T. kirilowii*, the continuous development of galls indicates that the RKNs successfully overcame these defense responses, ultimately resulting in a successful infection. Therefore, our findings reveal the complexity of host molecular response mechanisms within a susceptible context. Notably, the significant alterations in the phenylpropanoid, flavonoid, and terpenoid biosynthetic pathways revealed in this study are core changes in *T. kirilowii*’s response to an RKN infection. These secondary metabolites are not only crucial components of the plant defense response, but many also serve as key bioactive compounds for *T. kirilowii*’s medicinal efficacy. Although this study did not directly measure these metabolite changes, the extensive transcriptional reprogramming suggests that RKN infection may indirectly influence the accumulation and composition of active compounds in medicinal parts by disrupting these biosynthetic pathways, potentially affecting *T. kirilowii*’s quality. This provides important molecular clues for subsequent studies investigating the impact of biotic stress on medicinal plant quality formation at the metabolomics level.

### 3.4. Transcription Factor Regulatory Networks

Transcription factors play a crucial role in plant responses to RKN infection. The MYB family strengthens cell walls by regulating phenolic metabolism and wall modification genes [47]. AP2/ERF factors act as key effectors in the JA/ET pathway, driving phytoalexin and PR gene expression [48]. NAC factors govern stress responses and cell fate, balancing defense activation and pathogen manipulation [49]. bHLH factors integrate internal and external signals to modulate JA signaling and secondary metabolism [50]. WRKY factors centrally regulate JA and SA pathways, activating defense-related genes [51,52]. Our findings demonstrate that *T. kirilowii*’s defense against RKNs involves the MYB, AP2/ERF, bHLH, NAC, and WRKY families, with additional screening identifying three key transcription factors. The TRINITY_DN4568_c0_g3 transcription factor, designated as the ethylene-responsive transcription factor RAP2-13-like, has been experimentally validated to play essential roles in regulating cytokinin- and ethylene-related defense genes during plant responses to biotic stress [53,54]. The TRINITY_DN9312_c0_g1 transcription factor, identified as Suppressor of gamma response 1-like, participates in stress responses and apoptosis regulation, potentially functioning to restrict nematode progression within vascular tissues [55,56]. TRINITY_DN2196_c1_g1, a transcription factor MYB59-like, has been demonstrated to play crucial roles in cell cycle regulation, root development, and metabolic control, with studies further revealing its inhibitory effects on syncytium formation or RKN development [57,58]. These hub transcription factors represent key molecular switches for understanding *T. kirilowii*’s defense potential and the nodes determining susceptibility.

## 4. Materials and Methods

### 4.1. Experimental Materials

The *T. kirilowii* seedlings were obtained through tissue culture. The RKN (*Meloidogyne incognita*) was purchased from Suixian Biological Testing Agricultural Development Company and subsequently propagated on tomato (*Solanum lycopersicum* L.) seedlings for propagation and storage [59].

### 4.2. RKN Incubation and Infection

RKN egg masses in sterile water were selected and incubated at 25 °C in darkness for 5 days. Subsequently, the hatched second-stage juveniles (J2) were collected and a suspension at a concentration of 1000 individuals per milliliter was prepared [29]. *T. kirilowii* seedlings with uniform growth status obtained from tissue culture were transplanted into seedling pots (10 cm × 10 cm × 7.5 cm) containing a sterilized substrate, with one plant per pot. The seedlings were cultivated in a greenhouse (25 ± 3 °C, 16 h light photoperiod). After 20 days of growth, RKN infection was performed. Each seedling received 1 mL of RKN suspension applied by pipette injection into the rhizosphere soil at depths of 2–5 cm. The control group received an equal volume of sterile distilled water. Each treatment comprised 30 replicates.

### 4.3. Sample Processing and Collection

*T. kirilowii* seedlings infected with RKNs were cultivated in a greenhouse (25 ± 3 °C, 16 h light photoperiod). Root samples were collected at 0 dpi, 3 dpi, 6 dpi, 12 dpi, and 24 dpi post-infection. The collected samples were divided into two groups: the first group was used to determine gall number, gall length, and gall width; and the second group was used for transcriptome sequencing. Three seedlings were randomly selected from each treatment to form one biological replicate, resulting in three replicates (9 seedlings per treatment). Samples for transcriptomic sequencing were rinsed with sterile water immediately after collection, rapidly frozen in liquid nitrogen, and stored at −80 °C [30].

### 4.4. RNA Extraction and Sequencing

Three independent root samples were collected at each infection time point. Total RNA was extracted from *T. kirilowii* root tissue using TRIzol^®^ reagent (Invitrogen, Carlsbad, CA, USA) according to the manufacturer’s protocol [29]. RNA integrity (RIN > 7.0) was assessed using an Agilent 5300 Bioanalyzer (Agilent Technologies, Santa Clara, CA, USA), and RNA quantification was performed with a NanoDrop ND-2000 spectrophotometer (Thermo Fisher Scientific, Wilmington, DE, USA) [60]. RNA purification, reverse transcription, cDNA library construction, and sequencing were performed by Shanghai Meiji Biotechnology Co., Ltd. (Shanghai, China) according to the manufacturer’s protocols. The Illumina Stranded mRNA Prep Kit (Illumina, San Diego, CA, USA) was used for library construction with 1 μg total RNA as the starting template. The specific steps were as follows: mRNA was enriched using Oligo(dT) magnetic beads, followed by fragmentation (fragment length approximately 100–200 bp). First-strand and second-strand cDNA synthesis was performed using six-base random primers and reverse transcriptase, followed by end repair, phosphorylation, and adapter addition according to the library construction protocol. Target fragments (300–400 bp) were enriched via magnetic beads, amplified through 10–15 PCR cycles, and quantified using the Qubit 4.0 Fluorometer System (Thermo Fisher Scientific, Waltham, MA, USA) [61]. Final sequencing was performed on an Illumina NovaSeq X Plus platform (Illumina, San Diego, CA, USA) in PE150 mode with NovaSeq reagents.

### 4.5. Quality Control and Denovo Assembly of Sequencing Data

Raw paired-end sequencing reads were trimmed and quality controlled using fastp software (v0.18.0) with default parameters [62]. De novo assembly of cleaned data was performed using Trinity v2.4 software [63]. To improve the assembly quality, all assembled sequences were filtered through CD-HIT (similarity threshold ≥ 95%) and TransRate and evaluated using BUSCO v5.0 to ensure the reference sequence quality for subsequent analyses [64,65,66]. Assembled transcripts were homologously aligned against NR, KOG, GO, Swiss-Prot, eggNOG, and KEGG databases using Diamond v2.0 (E-value < 1 × 10^−5^), followed by functional annotation [67,68].

### 4.6. Differentially Expressed Gene (DEG) Screening

Gene expression levels were estimated using Transcripts Per Million (TPM) values [69]. Differential gene expression analysis employed the DESeq2 method, screening DEGs based on the criteria |log2(FoldChange)| ≥ 1 and FDR < 0.05 [70].

### 4.7. GO and KEGG Pathway Enrichment Analysis

Cluster Profiler v4.0 software was employed for GO functional classification and KEGG pathway enrichment analysis of DEGs. Adjusted *p*-values < 0.05 were used as the threshold for significant enrichment, and enrichment bubble plots were generated [71,72].

### 4.8. Weighted Gene Co-Expression Network (WGCNA) Analysis

Genes with TPM values > 1 were selected for co-expression network analysis using the WGCNA R package (v1.72-5) [73]. First, the correlation coefficient matrix between genes was calculated, and the soft threshold power was determined using the dynamic cutting method. The soft threshold power was ultimately set to 14 (scaleless network topology fit index R^2^ > 0.8) to achieve a scale-free topology. Gene co-expression modules were identified via dynamic tree cutting, and gene information within each module was extracted. The co-expression module structure was visualized through hierarchical clustering and dendrograms [74]. Module connectivity and module eigenvalues (MEs) were calculated for genes. Pearson correlation analysis assessed the association strength between modules and phenotypic traits, including infection time, gall number, gall length, and gall width (|r| > 0.5 and *p* < 0.05 were considered significantly correlated).

### 4.9. Hub Gene Screening and Gene Network Construction

Interactions among DEGs were analyzed using the STRING v12.0 database [75]. Genes with a combined interaction score > 0.4 and a connectivity ranking within the top 30 in the modules were selected as candidate core genes. The selected candidate core genes were further analyzed using the CytoHubba plugin in Cytoscape v3.9.1 through 12 centrality algorithms (degree centrality, betweenness centrality, proximity centrality, clustering coefficient, effective centrality, minimal network centrality, maximal network centrality, radial centrality, stress centrality, strain centrality, bottleneck centrality, and eccentricity) [61,76,77]. The genes identified by ≥8 algorithms were defined as hub genes and visualized via Cytoscape.

### 4.10. Quantification and Validation of Gene Expression Levels

To validate the accuracy of the RNA-seq results, 12 DEGs were randomly selected for validation. All primers are listed in Table S1. The obtained RNA was reverse transcribed into cDNA using a reverse transcription kit (Novogene Co., Ltd., Beijing, China). Using the synthesized cDNA as a template, qRT-PCR analysis was performed with the SYBR qPCR Kit (Vazyme Biotech Co., Ltd., Nanjing, China) on a QuantStudio 5 Thermal Cycler (Thermo Fisher, Waltham, MA, USA). *T. kirilowii* 18S rRNA was used as the reference gene. Gene expression levels were calculated using the 2^−ΔΔCT^ method, with standard error calculated based on three biological replicates.

### 4.11. Data Analysis and Statistics

Data were maintained in Excel 2016 and analyzed using SPSS Statistics 19 (Chicago, IL, USA). Differences among the time points of gall phenotypic traits were determined by one-way ANOVA, followed by Duncan’s multiple range test.

## 5. Conclusions

This study constructed a temporal dynamic transcriptomic profile of *T. kirilowii* and deeply analyzed the molecular mechanisms underlying its response to RKN infection. Results indicate that *T. kirilowii* roots exhibit progressively deteriorating phenotypes over the infection duration, accompanied by substantial differential gene expression. Transcriptome analysis revealed that the enriched pathways centered on disease resistance, defense responses, metabolic reprogramming, hormone signaling, and MAPK pathways play crucial roles in *T. kirilowii*’s response to RKN infection. Gene weighted co-expression network analysis and CytoHubba analysis identified four key modules and 33 hub genes. These genes, particularly the transcription factors among them, constitute central nodes in the response network; however, their precise functions in the *T. kirilowii*–RKN interaction require further validation through genetic experiments. This study elucidated the response characteristics of *T. kirilowii* to RKN infection, revealed its multi-level regulatory network for defense, and deepened the understanding of the molecular mechanisms; the identified hub genes potentially serve as important targets for resistance gene discovery. It also contributes to a deeper comprehension of the molecular interactions between medicinal plants and RKNs, providing a scientific basis for dissecting resistance mechanisms in medicinal plants and developing novel strategies for RKN control.

## Figures and Tables

**Table 1:** Transcriptome sequencing results.

Sample	Raw Reads (bp)	Clean Reads (bp)	Q30 (%)	GC Content (%)	Mapped Ratio (%)
0 dpi_1	42,310,432	42,074,692	95.91	44.01	86.57
0 dpi_2	39,778,516	39,561,214	95.65	43.98	86.59
0 dpi_3	42,449,858	42,203,302	95.85	44.23	87.25
3 dpi_1	40,940,936	40,702,856	95.69	44.34	86.78
3 dpi_2	43,478,764	43,243,934	95.79	43.93	86.39
3 dpi_3	42,436,186	42,220,302	95.64	44.36	86.89
6 dpi_1	45,972,092	45,733,338	95.81	44.2	86.60
6 dpi_2	41,516,762	41,289,510	95.68	44.28	87.16
6 dpi_3	42,471,732	42,252,240	95.73	44.25	87.61
12 dpi_1	42,185,042	41,964,686	95.8	44.44	87.12
12 dpi_2	44,182,840	43,942,854	95.76	44.17	86.75
12 dpi_3	42,189,546	41,964,990	95.69	44.43	86.81
24 dpi_1	45,146,812	44,920,136	95.53	44.53	86.56
24 dpi_2	47,777,050	47,544,140	95.66	44.59	86.66
24 dpi_3	47,332,584	47,074,882	95.57	44.55	86.65

**Table 2:** Identification of hub genes in key modules.

Module	Gene ID	Annotation
Yellow	TRINITY_DN5608_c0_g1	Ammonium transporter 2
	TRINITY_DN2459_c0_g1	Metacaspase-1
	TRINITY_DN2708_c0_g1	Phospholipase D beta 1
	TRINITY_DN1684_c0_g4	Cinnamoyl-CoA reductase-like SNL6
	TRINITY_DN20111_c0_g1	Potassium transporter 6
	TRINITY_DN4568_c0_g3	ethylene-responsive transcription factor RAP2-13-like
	TRINITY_DN2348_c0_g1	Cysteine-rich receptor-like protein kinase 43
	TRINITY_DN9312_c0_g1	Suppressor of gamma response 1-like
	TRINITY_DN2218_c0_g1	IQ domain-containing protein IQM4
	TRINITY_DN931_c0_g1	Helicase-like transcription factor CHR28
Blue	TRINITY_DN15408_c0_g1	Cyclin-A3-1
	TRINITY_DN6220_c0_g4	Uncharacterized protein LOC120081950
	TRINITY_DN37545_c0_g1	Uncharacterized protein LOC120068455
	TRINITY_DN16861_c0_g2	Cell division cycle-associated 7-like protein
	TRINITY_DN3759_c0_g1	Chaperonin CPN60, mitochondrial
	TRINITY_DN12594_c0_g2	Mitochondrial import inner membrane translocase subunit TIM8
	TRINITY_DN9745_c0_g2	hypothetical protein SDJN03_01940
Brown	TRINITY_DN5555_c0_g1	Probable BOI-related E3 ubiquitin-protein ligase 3
	TRINITY_DN12135_c0_g2	Hypothetical protein SDJN03_16570
	TRINITY_DN25049_c0_g1	CASP-like protein 2B1
	TRINITY_DN5047_c0_g1	Probable amidase At4g34880
	TRINITY_DN7761_c1_g1	Uncharacterized protein LOC111465553 isoform X2
	TRINITY_DN25814_c0_g1	Glycine-rich domain-containing protein 1
	TRINITY_DN2196_c1_g1	Transcription factor MYB59-like
	TRINITY_DN19012_c0_g3	WAT1-related protein At5g07050
	TRINITY_DN4616_c0_g1	Probable inactive shikimate kinase like 1, chloroplastic
	TRINITY_DN7559_c0_g1	Protein FAR-RED-ELONGATED HYPOCOTYL 1-LIKE
Turquoise	TRINITY_DN8171_c0_g1	Glutamine synthetase
	TRINITY_DN6343_c0_g1	Leucine aminopeptidase 1
	TRINITY_DN12145_c0_g1	Translocon-associated protein subunit alpha
	TRINITY_DN8063_c0_g1	Conserved regulator of innate immunity protein 3
	TRINITY_DN7981_c0_g1	Rab GDP dissociation inhibitor alpha
	TRINITY_DN4692_c0_g1	Proteasome subunit alpha type-2

## Data Availability

The sequence data were deposited in the NCBI Short Read Archive (SRA) database under the BioProject ID: PRJNA1395323.

## Supplementary Materials

The following supporting information can be downloaded at https://www.mdpi.com/article/10.3390/ijms27031594/s1.

## Abbreviations

The following abbreviations are used in this manuscript:

RKN	Root-knot nematode
DEG	Differentially expressed gene
WGCNA	Weighted gene co-expression network analysis
GO	Gene ontology
KEGG	Kyoto Encyclopedia of Genes and Genomes
TPM	Transcripts Per Million
qRT-PCR	Quantitative real-time polymerase chain reaction
